# Digital Health Skillsets and Digital Preparedness: Comparison of Veterans Health Administration Users and Other Veterans Nationally

**DOI:** 10.2196/32764

**Published:** 2022-01-28

**Authors:** Charlie Wray, Janet Tang, Amy Byers, Salomeh Keyhani

**Affiliations:** 1 Section of Hospital Medicine San Francisco Department of Veterans Affairs Medical Center San Francisco, CA United States; 2 Department of Medicine University of California, San Francisco San Francisco, CA United States; 3 Division of Mental Health Services San Francisco Department of Veterans Affairs Medical Center San Francisco, CA United States; 4 Department of Psychiatry and Behavioral Health Sciences University of California, San Francisco San Francisco, CA United States

**Keywords:** digital health literacy, Veterans Health Administration, health care, telemedicine, veterans, digital tools, social risk factors, digital uptake

## Abstract

**Background:**

As health care systems shift to greater use of telemedicine and digital tools, an individual’s digital health literacy has become an important skillset. The Veterans Health Administration (VA) has invested resources in providing digital health care; however, to date, no study has compared the digital health skills and preparedness of veterans receiving care in the VA to veterans receiving care outside the VA.

**Objective:**

The goal of the research was to describe digital health skills and preparedness among veterans who receive care within and outside the VA health care system and examine whether receiving care in the VA is associated with digital preparedness (reporting more than 2 digital health skills) after accounting for demographic and social risk factors.

**Methods:**

We used cross-sectional data from the 2016-2018 National Health Interview Survey to identify veterans (aged over 18 years) who obtain health care either within or outside the VA health care system. We used multivariable logistic regression models to examine the association of sociodemographic (age, sex, race, ethnicity), social risk factors (economic instability, disadvantaged neighborhood, low educational attainment, and social isolation), and health care delivery location (VA and non-VA) with digital preparedness.

**Results:**

Those who received health care within the VA health care system (n=3188) were younger (age 18-49 years: 33.3% [95% CI 30.7-36.0] vs 24.2% [95% CI 21.9-26.5], *P*<.01), were more often female (34.7% [95% CI 32.0-37.3] vs 6.6% [95% CI 5.5-7.6], *P*<.01) and identified as Black (13.1% [95% CI 11.2-15.0] vs 10.2% [95% CI 8.7-11.8], *P*<.01), and reported greater economic instability (8.3% [95% CI 6.9-9.8] vs 5.5% [95% CI 4.6-6.5], *P*<.01) and social isolation (42.6% [95% CI 40.3-44.9] vs 35.4% [95% CI 33.4-37.5], *P*<.01) compared to veterans who received care outside the VA (n=3393). Veterans who obtained care within the VA reported more digital health skills than those who obtained care outside the VA, endorsing greater rates of looking up health information on the internet (51.8% [95% CI 49.2-54.4] vs 45.0% [95% CI 42.6-47.3], *P*<.01), filling a prescription using the internet (16.2% [95% CI 14.5-18.0] vs 11.3% [95% CI 9.6-13.0], *P*<.01), scheduling a health care appointment on the internet (14.1% [95% CI 12.4-15.8] vs 11.6% [95% CI 10.1-13.1], *P*=.02), and communicating with a health care provider by email (18.0% [95% CI 16.1-19.8] vs 13.3% [95% CI 11.6-14.9], *P*<.01). Following adjustment for sociodemographic and social risk factors, receiving health care from the VA was the only characteristic associated with higher odds (adjusted odds ratio [aOR] 1.36, 95% CI 1.12-1.65) of being digitally prepared.

**Conclusions:**

Despite these demographic disadvantages to digital uptake, veterans who receive care in the VA reported more digital health skills and appear more digitally prepared than veterans who do not receive care within the VA, suggesting a positive, system-level influence on this cohort.

## Introduction

In recent years, and further expedited by the COVID-19 pandemic, health systems have shifted greater amounts of health care from in-person to digital-based care. The gathering of online information, use of mobile apps, and virtual-based patient-provider interactions require greater digital knowledge and skills from health care consumers. These abilities, termed digital health literacy, refer to the “set of skills and knowledge that are essential for productive interactions with technology-based health tools” [1]. An individual’s digital health skillset remains important as recent studies have shown that individuals’ self-perceived skills to use online information may affect their health and quality of their health care and that a lack of such skills may lead to adverse clinical outcomes [1-3]. Prior work shows that multiple individual-level factors, including age, race/ethnicity, and social risk factors such as income, education, and marital status, influence one’s overall digital health literacy [2,4-6]. While digital health literacy frequently describes the individual skills or experiences a consumer may have, the degree to which an individual may be able to meaningfully engage digitally based (eg, digitally prepared) health care is lacking. Digital preparedness can be thought of as having sufficient digital skills and experiences using digital tools to support digital-based care.

The health care system in which a person receives care may impact an individual’s digital health skillset and, thus, their digital preparedness in important ways. For instance, the Veterans Health Administration (VA), which cares for more than 9 million individuals and is the largest integrated health care system in the United States, has used technology-based interventions to improve patient access and outcomes. Because many veterans who receive care from the VA reside in rural locations, telemedicine and other asynchronous digital modalities are commonly used to provide health care within the VA [7]. Even prior to the pandemic, the VA was seen as a leader in the implementation and use of digital health interventions [8]. While prior work has found that most veterans have basic digital access (ie, own a digital device) and digital literacy (ie, ability to use the internet) to engage in digital-based care [9,10], to our knowledge, no study has compared the digital health skillset and preparedness of consumers of different health systems, as well as individual-level factors such as age, sex, race, ethnicity, and social factors (eg, economic instability, education attainment, and social isolation). Due to the VA’s history and focus on the use of digital care, we hypothesize that individuals who obtain their health care from the VA may have a greater digital health skillset and higher rates of digital preparedness (ie, reporting more than 2 digital health skills) than those who receive care outside the VA health care system.

To examine this, we used the National Health Interview Survey (NHIS) to first describe the digital health skillset and examine sociodemographic and social risk factors associated with digital preparedness among veterans who receive care from the VA in comparison with veterans who receive care outside the VA health care system. Then, we determined whether receiving care in the VA is associated with digital preparedness independent of these demographic and social risk factors.

## Methods

### Data Source

We used cross-sectional data from the 2016-2018 NHIS, a nationally representative sample of noninstitutionalized individuals residing within the United States, conducted annually by the National Center for Health Statistics at the Centers for Disease Control and Prevention [11]. The NHIS uses computer-assisted personal interviewing to annually administer the survey and collect health-related information from respondents. During the assessed years, the unconditional final adult response rate ranged from 53.0% to 54.3%. This study used publicly available data and was exempt from institutional review board review.

### Analytic Sample

After limiting the sample to respondents aged over 18 years and excluding individuals with missing data (<3%), our analytic samples included 3188 veterans who obtain care in the VA (which included VA, TRICARE [health insurance for active-duty military], and CHAMP-VA [Civilian Health and Medical Program of the Department of Veterans Affairs]) and 3393 veterans who received care outside the VA. To create these cohorts, we first used the question, “Have you ever served on active duty in the US Armed Forces, military reserves, or National Guard?” to identify veterans from nonveterans. Next, we used the question, “What kind of health insurance or health care coverage do you have?” and identified those who are receiving health care within the VA by those who answered, “military health care (TRICARE, VA, CHAMP-VA)” and those who received health care outside the VA by those who answered any type of insurance (ie, private, Medicare, Medicaid, Medigap) other than “military health care.”

Because this identification method may lack specificity, we also performed a sensitivity analysis on a secondary cohort from a 2018 subpopulation who were given the question “Have you ever enrolled in or used VA health care?” We then performed the same analysis among individuals who responded “yes” to this question.

### Covariates and Social Risk Factors

In our analysis of digital preparedness, we included age, sex, race, ethnicity, and 4 social risk factors (economic instability, disadvantaged neighborhood, low educational attainment, and social isolation) known to impact an individual’s digital health skillset [2,4-6]. The NHIS questionnaires were assessed for questions that addressed any of the 4 social risk factors (Multimedia Appendix 1). Respondents were considered to have a social risk factor if they answered positively (eg, yes) to any question pertaining to any of the 4 social risk factors.

### Digital Health Skills and Digital Preparedness

We used the digital health questions available in the NHIS questionnaires. To assess an individual’s digital health skillset, we used the following question, “During the past 12 months, have you ever used computers for any of the following...?” with the following 4 subquestions: “...to look up health information on the internet,” “...to fill a prescription using the internet,” “...to schedule an appointment with a health care provider on the internet,” and “...to communicate with a health care provider by email.” We then summed the total number of digital health skills questions in which an individual stated they had performed that particular task in the prior 12 months to present a digital health skills count. Based on clinical experience, we then defined digital preparedness as having 2 or more of any of the aforementioned digital health skills, chosen to create a reasonable dichotomization between those who may have only completed one of these tasks and those who potentially partake in several different aspects of digital-based health care, thus being labeled as digitally prepared.

### Statistical Analysis

First, we calculated descriptive statistics for veterans who obtained care within the VA and veterans who obtained care outside the VA and included estimated proportions and their 95% confidence intervals. Next, we calculated the estimated prevalence of digital preparedness based on age, sex, race, ethnicity, and social risk among the 2 cohorts. Finally, we used logistic regression to estimate unadjusted and multivariable model odds ratios and 95% confidence intervals for each characteristic, with adjusted analysis controlling for all previously described covariates. We performed this analysis on the primary cohort and on the secondary, 2018 cohort for the sensitivity analysis. All descriptive and regression estimates accounted for the complex sampling design, and sampling weights were used to produce estimates representative of the US population. Given the unknown and complex pathways between the social risk factors, we assessed for multicollinearity between all variables before final modeling using variance inflation factor (threshold: >10) and tolerance values (threshold: <0.1) and found no evidence of collinearity. Statistical analyses were performed using SAS statistical software (version 9.4, SAS Institute Inc).

## Results

### Individual Characteristics

The analytic samples consisted of 3188 veterans who received care within the VA, and 3393 veterans who received care outside the VA. Those who received health care within the VA health care system were younger (age 18-49 years: 33.3% [95% CI 30.7-36.0] vs 24.2% [95% CI 21.9-26.5], *P*<.01), were more often female (34.7% [95% CI 32.0-37.3] vs 6.6% [95% CI 5.5-7.6], *P*<.01) and identified as Black (13.1% [95% CI 11.2-15.0] vs 10.2% [95% CI 8.7-11.8], *P*<.01), and reported greater economic instability (8.3% [95% CI 6.9-9.8] vs 5.5% [95% CI 4.6-6.5], *P*<.01) and social isolation (42.6% [95% CI 40.3-44.9] vs 35.4% [95% CI 33.4-37.5], *P*<.01) compared to veterans who received care outside the VA (Table 1).

Veterans who obtained care within the VA reported more digital health skills than those who obtained care outside the VA, endorsing greater rates of looking up health information on the internet (51.8% [95% CI 49.2-54.4] vs 45.0% [95% CI 42.6-47.3], *P*<.01), filling a prescription using the internet (16.2% [95% CI 14.5-18.0] vs 11.3% [95% CI 9.6-13.0], *P*<.01), scheduling a health care appointment on the internet (14.1% [95% CI 12.4-15.8] vs 11.6% [95% CI 10.1-13.1], *P*=.02), and communicating with a health care provider by email (18.0% [95% CI 16.1-19.8] vs 13.3% [95% CI 11.6-14.9], *P*<.01; Table 1).

**Table 1:** Sociodemographics and digital health skills among veteran respondents to the National Health Interview Survey 2016-2018.

			Veterans who receive care within the VA^a^ (n=3188), % (95% CI)		Veterans who receive care outside VA (n=3393), % (95% CI)		*P* value
**Age (years)**			—^b^		—		<.01
	18-49	33.3 (30.7-36.0)		24.2 (21.9-26.5)		—	
	50-64	24.3 (22.3-26.2)		22.8 (20.7-25.0)		—	
	65-74	25.1 (23.2-26.9)		26.7 (24.7-28.6)		—	
	≥75	17.2 (15.5-18.8)		26.1 (24.3-27.9)		—	
**Sex**			—		—		<.01
	Male	65.2 (62.6-67.9)		93.3 (92.3-94.4)		—	
	Female	34.7 (32.0-37.3)		6.6 (5.5-7.6)		—	
**Race and ethnicity**			—		—		<.01
	White	78.9 (76.7-81.2)		85.0 (83.0-86.9)		—	
	Black	13.1 (11.2-15.0)		10.2 (8.7-11.8)		—	
	Other	7.8 (6.3-9.3)		4.7 (3.5-5.8)		—	
	Hispanic	9.1 (7.4-10.8)		7.1 (5.4-8.7)		.08	
**Social risk factors**			—		—		—
	Economic instability	8.3 (6.9-9.8)		5.5 (4.6-6.5)		<.01	
	Disadvantaged neighborhood	17.7 (15.8-19.5)		16.5 (14.7-18.4)		.38	
	Low educational attainment	71.3 (69.1-73.4)		69.0 (66.7-71.3)		.12	
	Social isolation	42.6 (40.3-44.9)		35.4 (33.4-37.5)		<.01	
**Digital health skills^c^**			—		—		—
	Look up health information on the internet	51.8 (49.2-54.4)		45.0 (42.6-47.3)		<.01	
	Fill a prescription using the internet	16.2 (14.5-18.0)		11.3 (9.6-13.0)		<.01	
	Schedule a health care appointment on the internet	14.1 (12.4-15.8)		11.6 (10.1-13.1)		.02	
	Communicate with a health care provider by email	18.0 (16.1-19.8)		13.3 (11.6-14.9)		<.01	
**Digital skills count^d^**			—		—		<.01
	0	43.1 (40.6-45.7)		49.9 (47.5-52.3)		—	
	1	31.5 (29.2-33.9)		30.6 (28.6-32.6)		—	
	2	11.7 (10.11-13.2)		10.5 (9.06-11.9)		—	
	3	9.0 (7.63-10.4)		5.8 (4.8-6.9)		—	
	4	4.5 (3.55-5.5)		3.0 (2.1-3.8)		—	

^a^VA: Veterans Health Administration.

^b^Not applicable.

^c^Used a computer in the past 12 months for any of the following.

^d^Calculated by summing the total number of “yes” responses to the digital literacy questions.

### Prevalence of Digital Preparedness

**Figure 1:**
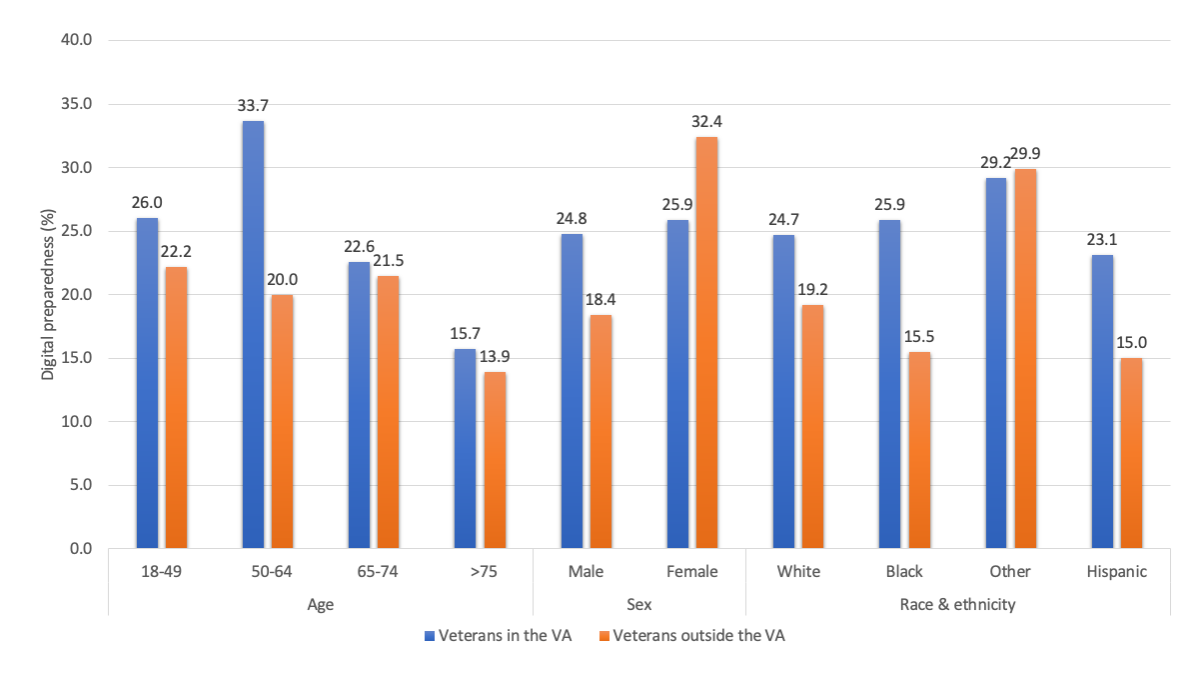
Prevalence of digital preparedness based on sociodemographics among veterans cared for within and outside the Veterans Health Administration. Digital preparedness is defined as having >2 “yes” responses to the four digital health skills questions. VA: Veterans Health Administration.

On average, veterans who received care in the VA were more digitally prepared (answering “yes” to more than 2 digital skills questions; mean 25.2%) than veterans who received care outside the VA (mean 20.8%). This difference was most pronounced among those who were middle aged (age 50-64 years; 33.7% vs 20%), males (24.8% vs 18.4%) and those who identified as Black (25.9% vs 15.5%). Among women, those who received care outside the VA reported more digital health skills than those who received care within the VA (32.4% vs 25.9%; Figure 1).

**Figure 2:**
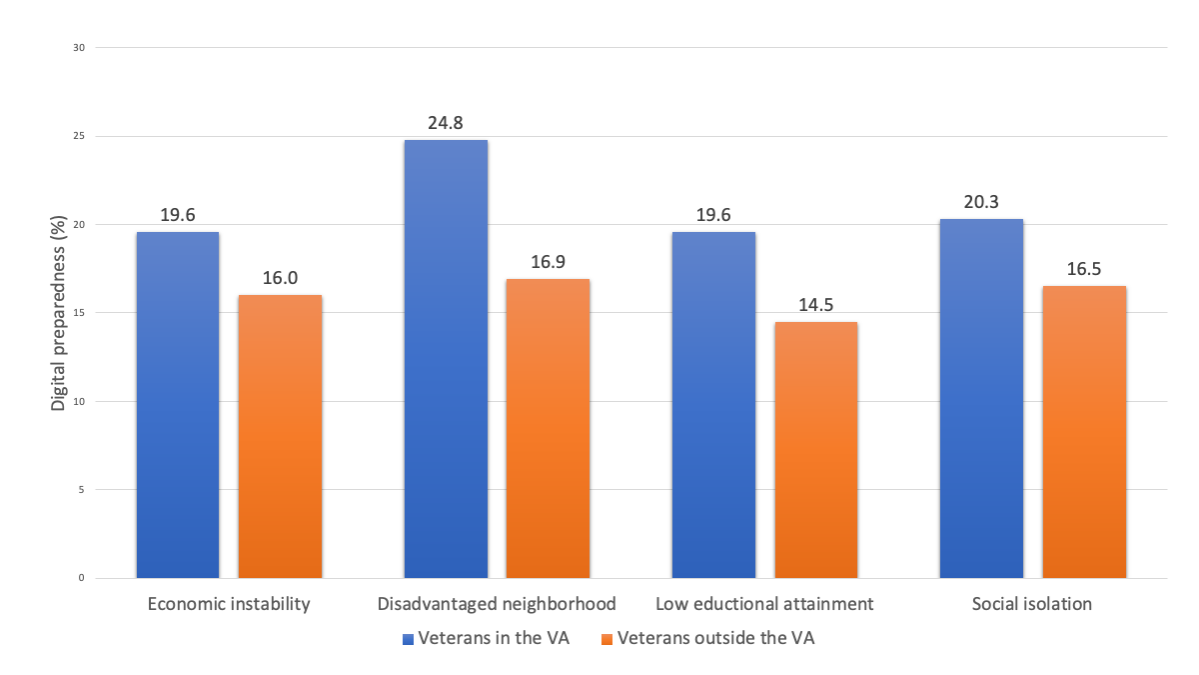
Prevalence of digital preparedness based on social factors among veterans cared for within and outside the Veterans Health Administration. VA: Veterans Health Administration.

Veterans who received care within the VA and who also reported economic instability (19.6%, 95% CI 13.4-25.8) and low educational attainment (19.6%, 95% CI 17.2-22.0) had the lowest prevalence of digital preparedness. Among veterans who received care outside the VA, social risk factors appeared to have a larger impact on the prevalence of digital preparedness (mean 15.5%) compared to those who received care within the VA (mean 21.2%), with individuals who reported low educational attainment (14.5%, 95% CI 12.6-16.5) having the lowest preparedness levels (Figure 2).

### Associations of Digital Preparedness

In unadjusted analysis, older age (over 75 years), low educational attainment, and social isolation were associated with significantly lower odds of being digitally prepared, while being female, identifying as a racial minority other than Black (eg, Asian, American Indian, Alaska Native, Native Hawaiian, or Pacific Islander), and receiving care at the VA were associated with higher odds of digital preparedness. In multivariable models, age over 75 years (adjusted odds ratio [aOR] 0.59, 95% CI 0.45-0.76), low educational attainment (aOR 0.40, 95% CI 0.34-0.48), and social isolation (aOR 0.78, 95% CI 0.66-0.92) remained significant negative predictors of digital preparedness. Receiving health care services from the VA was the only characteristic associated with higher odds (aOR 1.36, 95% CI 1.12-1.65) of being digitally prepared (Table 2).

**Table 2:** Sociodemographic, social risk factors, and health care systems association with digital preparedness^a^.

		Odds ratio of being digitally prepared^b,c^ (95% CI)	
		Unadjusted	Adjusted^d^
**Age (years)**			
	18-49	Reference	Reference
	50-64	1.12 (0.89-1.41)	1.16 (0.92-1.46)
	65-74	0.88 (0.70-1.11)	0.91 (0.72-1.16)
	≥75	0.53 (0.41-0.69)	0.59 (0.45-0.76)
**Sex**			
	Male	Reference	Reference
	Female	1.42 (1.19-1.70)	1.15 (0.95-1.40)
**Race and ethnicity**			
	White	Reference	Reference
	Black	0.95 (0.74-1.24)	0.87 (0.66-1.14)
	Other^e^	1.52 (1.09-2.12)	1.40 (0.97-2.01)
	Hispanic^f^	0.83 (0.55-1.26)^e^	0.72 (0.48-1.07)
**Social risk factors**			
	Economic instability	0.76 (0.56-1.05)	0.87 (0.62-1.22)
	Disadvantaged neighborhood	0.90 (0.72-1.12)	1.01 (0.80-1.26)
	Low educational attainment	0.39 (0.33-0.46)	0.40 (0.34-0.48)
	Social isolation	0.70 (0.60-0.82)	0.78 (0.66-0.92)
**Health care access**			
	Non-VA^g^ health care	Reference	Reference
	VA health care	1.40 (1.19-1.67)	1.36 (1.12-1.65)

^a^Missingness ranged from 1.2% to 3.1%.

^b^Digitally prepared is defined as having 2 or more “yes” responses to digital literacy questions.

^c^Reference is 0-1 “yes” responses to digital skills questions.

^d^Controlled for age, sex, race, ethnicity, and social risk factors.

^e^Other includes: Asian, American Indian, Alaska Native, Native Hawaiian, and Pacific Islander.

^f^Non-Hispanic is reference.

^g^VA: Veterans Health Administration.

In the sensitivity analysis among those who answered “yes” to “Have you ever enrolled in or used VA health care?” we found similar results. In adjusted analysis, receiving health care from the VA (aOR 1.33, 95% CI 1.04-1.69) was associated with digital preparedness (Multimedia Appendix 2).

## Discussion

### Principal Findings

In this national survey assessment of US veterans, we found that despite demographic and social disadvantages to digital uptake, veterans who received care in the VA reported more digital health skills and were more likely to be digitally prepared (defined as having more than 2 digital literacy skills) compared to veterans who did not receive care within the VA. While previous work has highlighted individual-level factors that can affect digital skills, to our knowledge, this is the first study to assess how the health care system in which an individual receives care may influence an individual’s preparedness to use digital-based care. There are several potential explanations for these findings.

First, we found that veterans’ digital skillsets may be low regardless of where they obtained care (within or outside the VA health care system). These levels are similar to other national data which show that approximately one-fifth of all Americans may not have proper digital literacy skills [12]. We note that digital health skills may be low due to the sociodemographics associated with the veterans cared for by the VA, as it selectively cares for individuals who are older, less educated, more rural, and with lower socioeconomic status [13]—all factors known to be associated with lower digital health literacy [4,14,15]. Despite these demographic disadvantages to digital uptake, veterans who receive care in the VA appear to have more digital health skills and be more digitally prepared than veterans who do not receive care within the VA, suggesting a positive, system-level influence on these individuals. We highlight potential age and gender differences between our survey respondents, who appear to be younger than the general veteran population (median age 57.5 [SD 15.2] years) and disproportionately female (median 11.5% among VA users and 8.8% among non-VA users) when compared to known VA demographics [16,17].

We hypothesize that the differences in digital skillsets and preparedness may be a product of the VA’s historical use of telemedicine and digital-based tools. Beginning in 1994, the VA began a progressive uptake and use of telemedicine, with early phases characterized by local innovations and pilot studies centered around telehealth delivery [18]. A second phase of the VA’s dissemination and use of telehealth modalities began in 2004 and centered around systems approaches that supported early adoption of telemedicine and created national clinical, technological, and business foundations for the VA’s developing telemedicine platforms [18]. The growth in telemedicine use over the subsequent two decades led the VA to report that in 2016, 12% of all veterans had received some of their care through telemedicine modalities [19], while fewer than 1% of Medicaid and rural Medicare beneficiaries used telehealth services during the same time period [20,21]. In recent years, other large, integrated health care systems such as Kaiser Permanente have placed a large emphasis on the use of telemedicine to provide access to care for its constituents, although it is unclear how such efforts have impacted their constituents’ digital health skills [22].

In addition to its early use of telemedicine, the VA was an early adopter in using on-demand tools, mobile apps, and other forms of digital outreach to connect with the individuals it serves [23]. For example, in 2010, the VA was the first health care system to institute the Blue Button program, an online health portal that allows users direct access to their health data [24]. Additionally, in 2016, the VA was one of the first health care systems to perform mass distribution of video-enabled tablets to at-risk populations as a means of improving access to care [25]. We note that the impact of telemedicine and these programs may be amplified by veterans’ rural demographics (approximately 25% reside in rural areas) [26], and thus encouraging and potentially requiring greater use of these digital resources than the general public.

The nature of the digital health skills questions used in this assessment may also offer further insight into the degree of digital connectedness among these cohorts. While previous work around digital health primarily focuses on basic digital access parameters such as owning a computer or smartphone, access to broadband internet, and use of email [9,27,28], the questions used in this survey represent higher order or more active engagement of digital health services than simply having access to a digital device or internet. This component of our assessment is particularly interesting given that prior research has found that digital connectedness is more often associated with younger age, higher education, and better health status [29]. Our findings highlight that while veterans have access to digital tools (eg, on average 80% use the internet or own a private computer) [9], only 1 in 6 veterans who received care within the VA (and only 1 in 8 who received care outside the VA) use these skills to meaningfully engage the health care system (eg, fill a prescription, schedule a health care appointment, or communicate with a health care provider through a computer or the internet). These findings suggest that simply relying on questions that assess access technology or internet use to estimate digital literacy may overestimate the actual degree of digital health literacy among this group [30].

Improving digital skills and literacy will be an important topic for health care systems to address as use of digital-based technologies expands in the coming years. It is well known that to improve use of digital tools, health care must go beyond access alone and improve individuals’ digital and health knowledge, numeracy, navigability, communication, and decision-making skills [31]. Several strategies have been used to improve digital literacy, including collaborative learning (ie, interacting with others to improve an individual’s digital skillset), which was found to improve participants’ computer and web knowledge, digital self-efficacy, and overall literacy skills [4], whereas studies that provided tailored educational interventions significantly improved not just digital literacy skills but health outcomes such as blood pressure [32,33] and medication adherence [34,35]. Our findings along with a review of the literature [4] show that interventions targeted at older, more vulnerable populations may be highly impactful and needed as more care migrates to such modalities.

### Limitations

Our study has notable limitations. First, the categorization method used to classify respondents based on where they receive their health care could misclassify some individuals, as some veterans may have military health insurance but could be obtaining care outside the VA. We note that our findings did not change when we performed a sensitivity analysis on respondents who directly stated they had VA-based health care. Second, our definition of being digitally prepared may be overly strict and potentially overpenalizes our characterization of who is digitally prepared. Third, this study did not include other potential risk factors that may have direct or confounding effects on digital preparedness, such as secondary barriers to digital access—cognitive, psychosocial, or functional barriers (eg, visual impairment)—that may impede optimal uptake and use of digital resources. Fourth, our outcomes of interest (digital health skills and digital health preparedness) are based on self-report, which could be biased or incorrect, as prior work has shown that individuals can both over- and underestimate their digital skillset [2], although such misclassification would likely be similar for those who receive care within the VA and those who receive care outside the VA. Fifth, the use of the term computer in the survey question could be misleading and may underestimate the use of cellphones or other smart devices to accomplish the queried task. Finally, this survey was conducted in the years prior to the COVID-19 pandemic, after which health care systems rapidly adopted telemedicine and other digital care modalities, and thus our findings may not be representative of current digital health skillsets or preparedness levels.

### Conclusions

Veterans who obtain services within the VA report greater digital health skills and appear more prepared to engage with health providers through digital means compared to veterans who receive their care outside the VA health care system, despite a higher prevalence of risk factors known to negatively impact digital literacy. These findings suggests that while individual-level barriers to digital care exist, there may be system-level factors or influences that moderate such barriers among at-risk populations such as those served by the VA. As digital-based care becomes more prominent, future work should focus on what system-based interventions or programs are improving individuals’ digital skillsets and ability to engage through digital mechanisms.

## Appendix

Multimedia Appendix 1 Domains and specific questions of social risk.

Multimedia Appendix 2 Sensitivity analysis among 2018 respondents who stated they have enrolled in or used VA-based health care.

## Abbreviations

aOR: adjusted odds ratio
CHAMP-VA: Civilian Health and Medical Program of the Department of Veterans Affairs
NHIS: National Health Interview Survey
VA: Veterans Health Administration

## References

[1] Norman CD, Skinner HA (2006). eHealth literacy: essential skills for consumer health in a networked world. J Med Internet Res.

[2] van der Vaart R, Drossaert C (2017). Development of the digital health literacy instrument: measuring a broad spectrum of Health 1.0 and Health 2.0 skills. J Med Internet Res.

[3] Neter E, Brainin E (2012). eHealth literacy: extending the digital divide to the realm of health information. J Med Internet Res.

[4] Watkins I, Xie B (2014). eHealth literacy interventions for older adults: a systematic review of the literature. J Med Internet Res.

[5] Tennant B, Stellefson M, Dodd V, Chaney B, Chaney D, Paige S, Alber J (2015). eHealth literacy and Web 2.0 health information seeking behaviors among baby boomers and older adults. J Med Internet Res.

[6] Chesser A, Burke A, Reyes J, Rohrberg T (2016). Navigating the digital divide: a systematic review of eHealth literacy in underserved populations in the United States. Inform Health Soc Care.

[7] Adams SV, Mader MJ, Bollinger MJ, Wong ES, Hudson TJ, Littman AJ (2019). Utilization of interactive clinical video telemedicine by rural and urban veterans in the Veterans Health Administration health care system. J Rural Health.

[8] Kellermann AL, Jones SS (2013). What it will take to achieve the as-yet-unfulfilled promises of health information technology. Health Aff (Millwood).

[9] Duan-Porter W, Van Houtven CH, Mahanna EP, Chapman JG, Stechuchak KM, Coffman CJ, Hastings SN (2018). Internet use and technology-related attitudes of veterans and informal caregivers of veterans. Telemed J E Health.

[10] Cho AH, Arar NH, Edelman DE, Hartwell PH, Oddone EZ, Yancy WS (2010). Do diabetic veterans use the Internet? Self-reported usage, skills, and interest in using My HealtheVet Web portal. Telemed J E Health.

[11] NHIS: National Health Interview Survey.

[12] Nouri S, Adler-Milstein J, Thao C, Acharya P, Barr-Walker J, Sarkar U, Lyles C (2020). Patient characteristics associated with objective measures of digital health tool use in the United States: a literature review. J Am Med Inform Assoc.

[13] Wilson NJ, Kizer KW (1997). The VA health care system: an unrecognized national safety net. Health Aff (Millwood).

[14] McInnes DK, Li AE, Hogan TP (2013). Opportunities for engaging low-income, vulnerable populations in health care: a systematic review of homeless persons' access to and use of information technologies. Am J Public Health.

[15] Clare CA (2021). Telehealth and the digital divide as a social determinant of health during the COVID-19 pandemic. Netw Model Anal Health Inform Bioinform.

[16] US Census American Community Survey: veteran status.

[17] Meffert BN, Morabito DM, Sawicki DA, Hausman C, Southwick SM, Pietrzak RH, Heinz AJ (2019). US veterans who do and do not utilize Veterans Affairs health care services: demographic, military, medical, and psychosocial characteristics. Prim Care Companion CNS Disord.

[18] Darkins A (2014). The growth of telehealth services in the Veterans Health Administration between 1994 and 2014: a study in the diffusion of innovation. Telemed J E Health.

[19] VA Telehealth Services.

[20] Park J, Erikson C, Han X, Iyer P (2018). Are state telehealth policies associated with the use of telehealth services among underserved populations?. Health Aff (Millwood).

[21] Mehrotra A, Jena AB, Busch AB, Souza J, Uscher-Pines L, Landon BE (2016). Utilization of telemedicine among rural medicare beneficiaries. JAMA.

[22] Lee S (2015). E-health equality for all: Kaiser Permanente's efforts to identify and address digital health disparities. Kaiser Permanente Institute for Health Policy.

[23] DigitalVA.

[24] VA Blue Button: download my data.

[25] Jacobs JC, Blonigen DM, Kimerling R, Slightam C, Gregory AJ, Gurmessa T, Zulman DM (2019). Increasing mental health care access, continuity, and efficiency for veterans through telehealth with video tablets. Psychiatr Serv.

[26] VA Office of Rural Health: rural veterans.

[27] Whealin JM, Jenchura EC, Wong AC, Zulman DM (2016). How veterans with post-traumatic stress disorder and comorbid health conditions utilize ehealth to manage their health care needs: a mixed-methods analysis. J Med Internet Res.

[28] Record EJ, Medoff DR, Dixon LB, Klingaman EA, Park SG, Hack S, Brown CH, Fang LJ, Kreyenbuhl J (2016). Access to and use of the internet by veterans with serious mental illness. Community Ment Health J.

[29] Fox CS, Hwang S, Nieto K, Valentino M, Mutalik K, Massaro JM, Benjamin EJ, Murabito JM (2016). Digital connectedness in the Framingham Heart Study. J Am Heart Assoc.

[30] Levine DM, Lipsitz SR, Linder JA (2016). Trends in seniors' use of digital health technology in the United States, 2011-2014. JAMA.

[31] Conard S (2019). Best practices in digital health literacy. Int J Cardiol.

[32] Neafsey PJ, Anderson E, Peabody S, Lin CA, Strickler Z, Vaughn K (2008). Beta testing of a network-based health literacy program tailored for older adults with hypertension. Comput Inform Nurs.

[33] Bosworth HB, Olsen MK, Grubber JM, Neary AM, Orr MM, Powers BJ, Adams MB, Svetkey LP, Reed SD, Li Y, Dolor RJ, Oddone EZ (2009). Two self-management interventions to improve hypertension control: a randomized trial. Ann Intern Med.

[34] Ownby RL, Hertzog C, Czaja SJ (2012). Tailored information and automated reminding to improve medication adherence in Spanish- and English-speaking elders treated for memory impairment. Clin Gerontol.

[35] Noureldin M, Plake KS, Morrow DG, Tu W, Wu J, Murray MD (2012). Effect of health literacy on drug adherence in patients with heart failure. Pharmacotherapy.

