# Supplementary material for: Digital Health Skillsets and Digital Preparedness: Comparison of Veterans Health Administration Users and Other Veterans Nationally
Source: JMIR Form Res. 2022 Jan 28;6(1):e32764. doi: 10.2196/32764 (PMC8838565; doi:10.2196/32764)
Supplement: Multimedia Appendix 1 [file formative_v6i1e32764_app1.docx]

Multimedia Appendix 1

| Domains and Specific Questions of Social Risk |
| --- |
|  |
| **Economic Instability**  Welfare assistance, job placement in the past year  Cash assistance from state/county welfare  Unemployed  Ever applied for Social Security Income (SSI)  Subsidized rent  Worry about maintaining current standard of living  Worry about enough money for retirement  Worry about paying normal monthly bills  Worry about inability to pay rent, mortgage, or housing costs  Worry about making minimum payment on credit cards |
| **Disadvantaged Neighborhood**  People in your neighborhood do not help each other out  There are no people you can count on in your neighborhood  People in your neighborhood cannot be trusted  Do not live in a close-knit neighborhood |
| **Low Educational Attainment**  No college or graduate degree  Using your usual language, you have difficulty communicating |
| **Social Isolation**  Lives alone  Difficult to participate in social gatherings (clubs, parties)  Difficult to go shopping, movies, or sporting events  Delayed getting medical care due to lack of transportation |
