# Supplementary material for: Digital Health Skillsets and Digital Preparedness: Comparison of Veterans Health Administration Users and Other Veterans Nationally
Source: JMIR Form Res. 2022 Jan 28;6(1):e32764. doi: 10.2196/32764 (PMC8838565; doi:10.2196/32764)
Supplement: Multimedia Appendix 2 [file formative_v6i1e32764_app2.docx]

Multimedia Appendix 2. Sensitivity analysis among 2018 respondents who stated they have enrolled in or used VA-based health care.

|  | **Odds Ratio of being Digitally Prepared ^a,b^**  **(95% CI)** | |
| --- | --- | --- |
|  | **Unadjusted** | **Adjusted^c^** |
| **Age**  18-49  50-64  65-74  >75 | Reference  1.16 (0.85 - 1.51)  0.91 (0.65 - 1.17)  0.58 (0.42 - 0.61) | Reference  1.19 (0.99 - 1.56)  0.95 (0.81 - 1.26)  0.62 (0.41 - 0.86) |
| **Sex**  Male  Female | Reference  2.42 (1.71 - 3.44) | Reference  2.39 (1.67 – 3.43) |
| **Race and Ethnicity**  White  Black  Other^d^  Hispanic^e^ | Reference  1.05 (0.34 - 1.49)  1.02 (0.62 – 1.67)  0.90 (0.51 - 1.59)^e^ | Reference  0.99 (0.69 - 1.42)  0.97 (0.58 – 1.61)  0.94 (0.54 - 1.65) |
| **Social Risk Factors**  Economic instability  Disadvantaged neighborhood  Low educational attainment  Social isolation | 0.93 (0.59 - 1.48)  0.78 (0.58 - 1.05)  0.37 (0.29 - 0.47)  0.74 (0.58 - 0.93) | 1.18 (0.74 - 1.89)  0.88 (0.65 - 1.20)  0.39 (0.30 - 0.51)  0.77 (0.61 - 0.97) |
| **Health care access**  Non-VA Health Care  Veteran’s Health Affairs | Reference  1.34 (1.04 - 1.69) | Reference  1.33 (1.04 - 1.69) |

**Notes**:

^a^ digitally prepared is defined as having 2 or more “yes” responses to Digital Skills questions

^b^ Reference is 0-1 “yes” responses to Digital Literacy questions

^c^ controlled for age, sex, race, ethnicity, and social risk factors

^d^ Other includes: Asian, American Indian, Alaska Native, Native Hawaiian, Pacific Islander

^e^ non-Latino is reference

missingness ranged from 1.2-3.1%
